# Cybersickness and Its Severity Arising from Virtual Reality Content: A Comprehensive Study

**DOI:** 10.3390/s22041314

**Published:** 2022-02-09

**Authors:** Heeseok Oh, Wookho Son

**Affiliations:** 1 Department of Applied AI, Hansung University, Seoul 02876, Korea; 2 SW&Content Research Lab., ETRI, Daejeon 34129, Korea; whson@etri.re.kr

**Keywords:** virtual reality (VR), VR human factor, cybersickness analysis, VR cybersickness dataset

## Abstract

Virtual reality (VR) experiences often elicit a negative effect, cybersickness, which results in nausea, disorientation, and visual discomfort. To quantitatively analyze the degree of cybersickness depending on various attributes of VR content (i.e., camera movement, field of view, path length, frame reference, and controllability), we generated cybersickness reference (CYRE) content with 52 VR scenes that represent different content attributes. A protocol for cybersickness evaluation was designed to collect subjective opinions from 154 participants as reliably as possible in conjunction with objective data such as rendered VR scenes and biological signals. By investigating the data obtained through the experiment, the statistically significant relationships—the degree that the cybersickness varies with each isolated content factor—are separately identified. We showed that the cybersickness severity was highly correlated with six biological features reflecting brain activities (i.e., relative power spectral densities of Fp1 delta, Fp 1 beta, Fp2 delta, Fp2 gamma, T4 delta, and T4 beta waves) with a coefficient of determination greater than 0.9. Moreover, our experimental results show that individual characteristics (age and susceptibility) are also quantitatively associated with cybersickness level. Notably, the constructed dataset contains a number of labels (i.e., subjective cybersickness scores) that correspond to each VR scene. We used these labels to build cybersickness prediction models and obtain a reliable predictive performance. Hence, the proposed dataset is supposed to be widely applicable in general-purpose scenarios regarding cybersickness quantification.

## 1. Introduction

### 1.1. Motivation

To maximize the sensation of reality, visual content is developed not only for flat displays (including UHD 8K, IMAX, and stereoscopic 3D), but also for more expressive environments such as augmented-, virtual-, and mixed-reality (AR, VR, and MR). Among them, VR hardware development is the most promising. The VR industry is rapidly emerging, and the users expect an increasingly high quality of experience for VR services. Although the VR environment can deliver an improved sensation of reality to users, such forced artificial reality using a head-mounted display (HMD) may also induce *cybersickness*. The term cybersickness is a motion sickness-like experience in a virtual reality which is visually induced [1]. The general frequent symptoms caused by cybersickness are nausea, visual discomfort, and disorientation [2]. Cybersickness restricts the use of VR and various theories exist regarding its cause, but whose analysis has been limited to human factors, not VR content. A previous study has reported that 80% of people show symptoms of cybersickness within 10 min of becoming immersed in the VR environment [3].

In early studies, the physiological mechanism of motion sickness was analyzed from a neuroscientific approach. Reason and Brand [4] postulated that motion sickness was a self-inflicted maladaptation caused by internal- and intra-sensory conflicts between visual, vestibular, and somatosensory proprioceptors, which is the most widely accepted theory. Oman [5] expanded sensory conflict theory to include a dynamic model with mathematical description, where motion sickness was caused by the state of sensory rearrangement. However, sensory conflict theory cannot sufficiently explain some classes of motion sickness provoked by an optokinetic circular movement and a head orientation relative to the gravity vector variation [6]. Bles et al. [7] elaborated on this theory by employing a vector sum of gravity and inertial acceleration of semicircular canals and otolith organs in the vestibular system based on the sensed vertical, which is known as subjective vertical conflict theory. Bos et al. [8] established an advanced model, emphasizing the concept of the neural store in the subsequent central nervous system to account for expectation, where visual–vestibular interaction was assumed to be responsible for causing visually induced motion sickness regarding self-motion. According to this theory, the sensory expectation on the basis of prior experiences is also a kind of conflict, with discrepancies between multiple sensory inputs [1].

In our study, we deal with cybersickness arising from viewing HMD-based VR. Despite similar sickness symptoms, visually induced cybersickness in an artificial environment (e.g., stereoscopic 3D display and projection-based VR) is not due to a physical movement and an external perturbation, but rather is provoked mostly by exposure to visual movement. In this context, previous theories and their dynamics provide an insight for mathematical analysis of the psycho-physical responses of the human when experiencing VR, which also provides meaningful cues for a quantitative investigation of the relationship between perceptual characteristics and VR content attributes.

**Figure 1:**
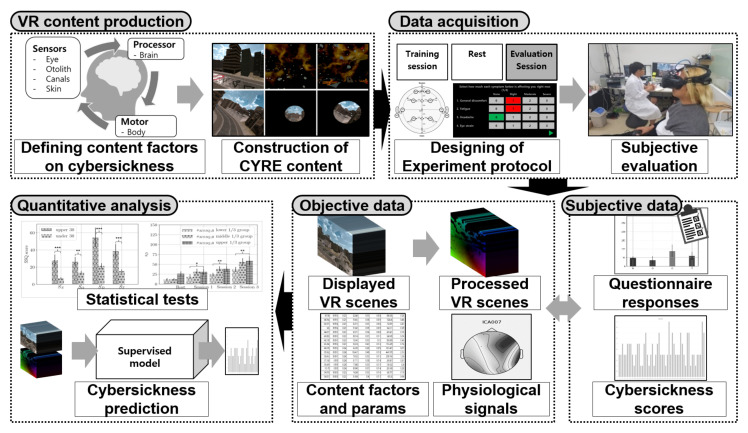
Overall framework for the construction of a dataset with subjective evaluation and the analysis of the degree of cybersickness. *, **, and *** represent that *p*-value is less than 0.05, 0.01, and 0.001, respectively.

First, we investigated which VR content factors dominate cybersickness, since previous studies focused on the hardware factor or fragmentary content attributes of cybersickness (detailed in Section 1.2). To conduct quantitative analysis on cybersickness in terms of VR content, we built a pipeline to collect reliable data and subjective opinions and to quantitatively investigate this large-scale dataset. Figure 1 depicts the overall framework of our study. First, the synthetic 3D VR content (cybersickness reference (CYRE) content) was produced to contain 52 content items (i.e., VR scenes) that correspond to various attributes that have been known or defined as provocative cybersickness factors with referring physiological motion sickness mechanisms. We designed a cybersickness evaluation protocol to obtain self-diagnosis responses to sickness symptoms using questionnaires, and the individuals’ characteristics, including sex, age, and motion sickness susceptibility, were also collected. In addition, we obtained a subjective sickness score for each scene using a graphical user interface during a rating procedure. Simultaneously, we acquired objective data, such as the VR scene displayed on HMD (i.e., rendered video), and biological signals, including electroencephalograph (EEG), electrocardiogram (ECG), and galvanic skin response (GSR). Based on data acquired from 154 valid participants, we performed statistical tests to identify a quantitative relationship between various factors and cybersickness. Our experimental design did not involve a cohort study, but rather was constructed in the form of changing only one dependent variable (i.e., a single content factor) while controlling the other variables.

In this paper, cybersickness severity is defined as the degree of cybersickness. In addition to the traditional statistical analysis of the collected data, we verified that we constructed a sufficient dataset, which enabled us to train the predictor which infers the objective value representing cybersickness severity based on the rendered VR scene. It is obvious that cybersickness severity was dealt with as a continuous value, not a binary decision (i.e., whether cybersickness is provoked or not) since the participants were asked to report the cybersickness score of 1–5 per VR scene. In general, to model an accurate cybersickness predictor to determine its severity, a large number of labels is required in a supervised manner. A number of labels was assigned to various measures by utilizing a subjective sickness score for 52 VR scenes; such supervisors enabled the trained model to extract the latent features that represent the severity of cybersickness. To verify the superiority of the constructed dataset in this study, we used several cybersickness prediction schemes, including deep-learning approaches. We showed their predictive performance in terms of their correlation to the participants’ opinions.

### 1.2. Related Work

Although a solid theoretical foundation and the approximated dynamic models that represent motion sickness have been introduced, there has not been a comprehensive study on cybersickness in terms of the VR content factor. Previous studies were interested in the binary decision of whether a certain VR factor provokes sickness or not. Moreover, most studies were hardware-oriented and considered parameters such as display type, display resolution, display field of view (FOV), and latency without considering the VR content [9,10,11,12,13,14,15,16,17].

#### 1.2.1. Content Factor-Based Cybersickness Analysis

Few studies dealt with the VR content factor following an ad hoc approach while only analyzing it in a qualitative manner. Hell and Argyriou [18] studied motion sickness depending on the content factors in a roller-coaster simulation. The stored parameters in the virtual environment were fed into the trained neural network model, where the considered attributes were biased to camera movements. Joseph et al. [19] focused on single-axis rotations and showed that roll and pitch rotations cause relatively higher motion sickness than yaw. Porcino et al. [20] emphasized the depth and calculated the importance of the virtual objects to mitigate cybersickness through a dynamic de-focus blurring. Fernandes and Feiner [21] investigated a trade-off relationship between cybersickness and a sense of presence with varying FOV. In a study with 30 volunteers, the results showed that the restricted FOV was effective to suppress the degree of cybersickness. So et al. [22] reported that VR navigation speed significantly affected the degree of vection, and the authors demonstrated that cybersickness is a type of vection-induced motion sickness. Chardonnet et al. [23] demonstrated that the distance from a virtual barrier and the choice of the navigation interface can mitigate cybersickness. Some studies suggested that using an independent visual background deployment as a frame reference is helpful to reduce the postural balance disturbance accompanied by cybersickness [24,25].

#### 1.2.2. Machine Learning for Quantifying Cybersickness

Apart from factor-based cybersickness analysis, some studies attempted to quantify cybersickness directly from VR content. Padmanaban et al. [26] designed a cybersickness prediction model based on the hand-crafted features (including relative depth and motion vectors) from $360^{\circ }$ stereoscopic video clips. The extracted features were regressed onto subjective sickness scores, which were obtained from the deployment of a post-questionnaire. Kim et al. [27,28] defined a concept of exceptional motion in VR content, and they predicted it by deploying an auto-encoder. The latent variables of their model were learned from easily downloadable $360^{\circ }$ scenes, and the results showed that the inferred exceptional motion was correlated with cybersickness. Lee et al. [29] modeled a cybersickness predictor for a $360^{\circ }$ stereoscopic video. The authors proposed a 3D convolutional kernel-based neural network to capture representative temporal abstractions related to cybersickness. Balasubramanian and Soundararajan [30] trained their model to predict the degree of visual discomfort based on 40 subjective ratings for viewing $360^{\circ }$ scenes.

Unfortunately, machine-learning approaches using $360^{\circ }$ VR content cannot provide any practical information to mitigate cybersickness. In other words, it is difficult to know which content factor has to be regulated. This limitation is mainly due to the impossibility of controlling the factors independently while capturing the VR scene and the unexplainable/uninvertible weighted parameters for each factor implied in the non-linearly trained model. Besides, another practical challenge is conducting a massive subjective test, owing to the limitations of employing questionnaires. The collection of subjective opinions using a post-questionnaire inevitably results in a lack of data-label pairs for supervised learning, because the response process is inconvenient, and only a single response (i.e., sickness score) can be obtained for several evaluated content items. In other words, the previous study had limitations because of the insufficient size of the dataset in terms of its supervised learning manner, and an additional data augmentation technique was required to model an accurate predictor [29].

#### 1.2.3. Quantitative Cybersickness Analysis via Biological Signal

Similar limitations regarding learning-based approaches also appeared in several studies based on biological measurements for quantifying cybersickness regarding the human factor. Guna and Gersak [31] observed ECG and GSR results of 26 participants who reported sickness after experiencing a VR environment, where only two $360^{\circ }$ VR clips were shown. Lin et al. [32] quantified cybersickness into three stages by categorizing the power of EEG and electro-oculography from 25 volunteers who participated in a $360^{\circ }$ VR experiment. Similarly, Islam et al. [33] quantified cybersickness severity using a neural network based on 31 participants’ physiological signals. In the most advanced study, Lin et al. [34] predicted the level of motion sickness from the EEG spectrum. A self-organizing neural fuzzy inference network was introduced to achieve it. However, their research mainly focused on car sickness in a virtually designed driving environment, rather than analyzing visually induced cybersickness arising from viewing VR. Consequently, since almost all experiments of previous researchers were limited to $360^{\circ }$ VR content, the factor-based analysis and the techniques for mitigating cybersickness in terms of VR content remain ungeneralized.

### 1.3. Contributions

The contributions of this study are as follows:

- We generated synthetic CYRE content: 52 VR scenes that represent different content factors associated with causing cybersickness.
- We designed a cybersickness evaluation protocol and obtained a number of subjective opinions from 154 participants in conjunction with objective data (e.g., rendered videos of VR scenes and biological signals) to construct a database.
- We quantitatively analyzed how various factors (e.g., content attributes, physiological responses, and individual characteristics including sex, age, and susceptibility to motion sickness) influence the severity of cybersickness.
- We constructed a number of data-label pairs (i.e., the number of scenes × the number of participants) for supervised learning.

## 2. Construction of CYRE Content

To conduct a subjective assessment and to collect data for cybersickness analysis depending on VR content factors, we created new CYRE content with various attributes. The main scenario of the generated content is flight navigation: 52 scenes with different factors, as shown in Table 1. Flight is a less common motion in VR than walking; however, several factors have been revealed in the past decades, and we selected controllable content factors to be quantitatively verified. Here, the scene-by-scene analysis of each content factor was performed, thus every VR scene pair exists in CYRE content as tabulated in Table 1 for single-factor analysis (i.e., only a single dependent variable exists while the other variables have to be controlled). The content was generated using the Unity 3D engine. The factors used in CYRE content are described as follows.

### 2.1. Background of Scene

**Figure 2:**
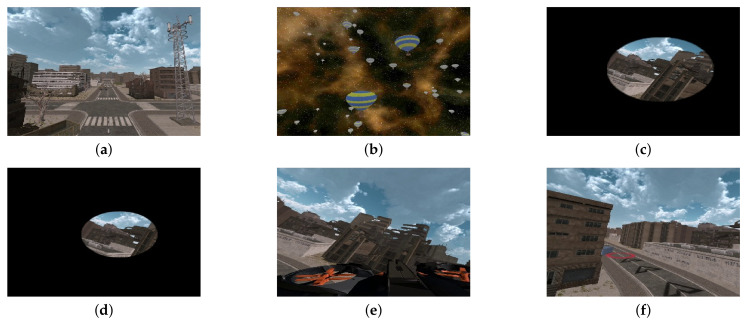
Examples of VR scenes in CYRE content: (**a**) 202, (**b**) S101, (**c**) S214, (**d**) S215, (**e**) S218, and (**f**) S001. Attributes of each scene are given in Table 1.

Regardless of the kind of VR content, a stationary object or background in the scene can suppress cybersickness [35]. In this context, CYRE content scenes include two background types: urban space and astrospace, as shown in Figure 2a,b. In the urban scenes, the terrain is always at the lower side of the viewer’s sight, and it can be regarded as a strong self-motion perception cue, which is unseen in the astrospace scenes. The urban scenes also contain several textured objects (e.g., buildings, bridges, and trees); their higher spatial complexity may provoke cybersickness (compared with the astrospace scenes) [36].

### 2.2. Camera Movement

Moving visual stimuli without physical motion induces illusory self-motion (i.e., vection) within virtual environments [37]. The lack of other sensory inputs for self-motion perception (i.e., vestibular and somatosensory stimuli) causes multi-sensory conflict that leads to severe cybersickness [38,39]. In this context, the camera movements in a VR scene are deeply related to the moving visual stimuli, which are key factors for analyzing cybersickness [40]. CYRE content includes two types of camera movement: simple and complex.

#### 2.2.1. Simple Movement

Some generated scenes include single-camera movements, i.e., rotations (i.e., yaw $r_{y}$, roll $r_{r}$, and pitch $r_{p}$) and translations (i.e., forward $t_{f}$, backward $t_{b}$, and lateral $t_{l}$). In particular, the upward, $t_{u}$, and downward, $t_{d}$, translations are also included in CYRE content to determine the effect of vertical movements on cybersickness. Each scene was produced to compare the influence of different primary movements upon the degree of cybersickness.

#### 2.2.2. Complex Movement

In general, a complex visual motion causes more severe cybersickness; however, it is non-linearly proportional to its degree [41,42]. In CYRE content, 36 scenes have dual- or triple-axis dynamic rotations combined with translations.

#### 2.2.3. Translation Acceleration

Acceleration is highly associated with the vector sum of gravity and inertial acceleration, and the unexpected, when sensed vertically by optokinetic signals, directly affects motion sickness [8,43,44]. Based on this, some scenes in CYRE content have acceleration for translation movements whose rates were ∼160% (i.e., the translation speed gradually increased up to 160% of the initial translation speed.). Thus, the degree of cybersickness could be analyzed by comparing these scenes with the other scenes that have a constant velocity (e.g., S110 and S116).

#### 2.2.4. Translation Speed

Beyond acceleration, Singla et al. showed that camera translation speed is also correlated to cybersickness [13]. CYRE content includes scenes with both moderate (4 m/s) and relatively fast (9.2 m/s) camera translation movements. Note that the value of speed is the Unity3D parameter (in camera coordinate), thus the perceived speed by humans (in the world coordinate) may be different due to the viewing geometry. All transitions have the same speed for the complex movement scenes. The purpose is to quantitatively analyze the relationship between the degree of cybersickness and the navigating speed. Note that our study only considers the comparison of the participants’ cybersickness corresponding to translation speed without examining rotation speed.

### 2.3. FOV

In general cases of VR environments, the internal FOV is characterized by the graphic generation engine, while the external FOV is determined by the HMD specification and the viewing geometry [45]. It is well known that a smaller external FOV can reduce cybersickness [21,46]. However, it is difficult to reflect external FOV as a VR content factor, because it can depend heavily on the display hardware. To analyze the effect of internal FOV on cybersickness, CYRE content includes three different sizes of internal FOV: small ($30^{\circ }$), middle ($45^{\circ }$), and large ($90^{\circ }$) FOVs, as shown in Figure 2c,d.

### 2.4. Frame Reference

A frame reference is strong evidence for consistent inertial sensing of when the rest regions are heavily influenced by the moving background [47]. In CYRE content, the head of the flight vehicle is seen as a frame reference at the lower side of a scene (Figure 2e, which is intended to provide similar effects to a “virtual nose” in the first-person-perspective VR content [35].

### 2.5. Duration (Path Length)

Previous studies indicated that the path length and duration of a VR experience could increase cybersickness [41,48,49]. This factor is also believed to generate CYRE content, and the length of the visual stimulus was determined by referring to previous studies [50,51]. The scenes have different lengths, which are characterized by the extended navigation routes. In other words, some of the scenes require a longer VR experience time (24 s) than the others (13 s), because their endpoint is far from the starting point. Note that a termination criterion of scenes S001–S008 is the ability of the users to reach the endpoint independently (S007 and S008 have a longer route than S001–S006); thus, the average experience times of all the participants are shown in Table 1.

### 2.6. Controllability

According to the sensory conflict theory, a neurological reflex exists in the perceptual mechanism when the human prepares to an anticipated visual motion [8]. In other words, an unexpected visual motion initiated by a passive (i.e., without control) camera movement contributes to cybersickness [52]. CYRE content includes both controllable and uncontrollable scenes in the same environment. In the controllable scenes, the flight vehicle movement direction can be freely controlled following a user’s intention. Additional navigation information is displayed graphically to keep the user on the route and to show the endpoint, as shown in Figure 2f.

## 3. Subjective Evaluation and Data Acquisition

### 3.1. Protocol Design

**Figure 3:**
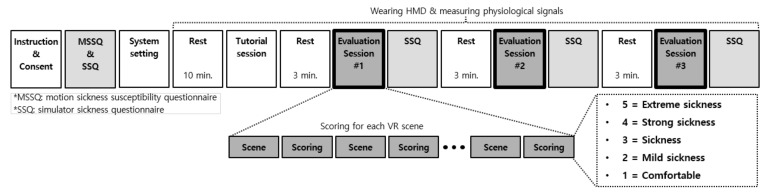
Designed protocol for subjective cybersickness evaluation.

Both objective data (i.e., scene parameters, rendered VR scenes, and physiological measures) and subjective data (opinions on the degree of cybersickness) have to be collected when the participants view the constructed CYRE content. Towards this, we designed a subjective evaluation protocol. An overall procedure is shown in Figure 3, and the detailed descriptions for each step are following.

#### 3.1.1. Tutorial Session

Before the evaluation, we conducted a tutorial session. The participants were introduced to the evaluation process and the degrees of cybersickness. During the instruction, six VR scenes were shown, broadly spanning the range of VR factors. The tutorial session helped to collect more reliable data in two ways. First, the participants were familiarized with the interactive controlling method used in this experiment, which helped to avoid a potentially unwanted stress factor (e.g., stress about not being able to control navigating at will). Second, the tutorial helped to reduce the biased subjective opinion for inexperienced content, due to the human’s psychological expectations that lead to a self-hypothesis, known as “experimental demand characteristics” [53].

#### 3.1.2. Evaluation Session

As the bold-framed boxes in Figure 3 show, the overall subjective evaluation procedure was divided into three evaluation sessions. As shown in Table 1, there are threecategories, $C_{\mathrm{CU}}$, $C_{\mathrm{UA}}$, and $C_{\mathrm{UU}}$, for representing the controllable urban scenes, uncontrollable astrospace scenes, and uncontrollable urban scenes, respectively. Their durations are 145, 234, and 371 s, respectively. The goal of our research is to investigate the relationship between the VR content factor and cybersickness, thus the temporal effects of cybersickness including accumulation and adaptation were out of the study scope [54]. To cope with this critical problem, we shuffled the order of the scene categories when assigning them to each evaluation session for each participant, and the order of the corresponding VR scenes (i.e., 8, 18, and 26 scenes for $C_{\mathrm{CU}}$, $C_{\mathrm{UA}}$, and $C_{\mathrm{UU}}$, respectively) was also randomly shuffled [55,56]. Such shuffling methodology can facilitate a relative comparison between each scene, and this would not affect the experiment results since each VR scene was scored individually (at a scene-level, each participant experiences the same VR scene). Here, the analysis of individual characteristics in Section 4.3 was performed based on each scene category, but whose controlled attributes were the same for each participant and only their displayed order was different. After each evaluation session and response to the corresponding questionnaires, rest periods of 3 min were inserted to minimize the accumulated VR sickness [50,57]. During the rest periods, a monotonous scene with a cross mark at the center of the frame was displayed to avoid the loss of the viewer’s focus.

#### 3.1.3. Questionnaires

Before viewing VR content, participants were asked to fill the motion sickness susceptibility questionnaire (MSSQ) [58] and the simulator sickness questionnaire (SSQ) [2], which are the traditional questionnaires used by motion-sickness-related researchers [59,60,61], as depicted in Figure 3. Such pre-assessment reflects the subjective opinions which lie on a neutral state. These responses could be compared with those acquired after the VR experience (in Section 4.3.3).

Originally, MSSQ was designed for self-diagnosis, i.e., to determine how much a participant is susceptible to motion sickness. MSSQ consists of two sections: Section A is for the motion sickness experience in childhood, and Section B is for the last 10 years. By a weighted summation, the motion sickness susceptibility score $s_{\mathrm{MSSQ}}$ is calculated as [58]

(1) $$s_{\mathrm{MSSQ}}=s_{\mathrm{MSSQ},A}+s_{\mathrm{MSSQ},B},$$

where $s_{\mathrm{MSSQ},A}$ and $s_{\mathrm{MSSQ},B}$ represent the scores of MSSQ Sections A and B.

SSQ rates the levels of 16 symptoms of motion sickness using a four-point scale (ranging from zero to three). SSQ evaluates the degree of three general classes of motion sickness: nausea, oculomotor, and disorientation. The scores of nausea $s_{N}$, oculomotor $s_{O}$, and disorientation $s_{D}$ are calculated from a selective sum of the levels of 16 symptoms, and a total score of motion sickness $s_{T}$ is the scaled sum of all subjective scores of symptoms belonging to the three general classes [2]. It is worth noting that $s_{N}$, $s_{O}$, $s_{D}$, and $s_{T}$ are unnormalized values, which can be relatively compared.

**Figure 4:**
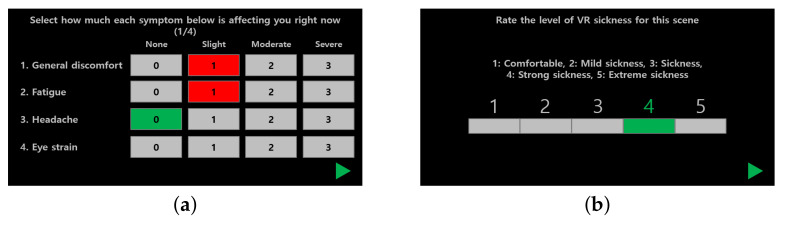
Graphically generated supporting content for subjective evaluation. (**a**) Graphical user interface for answering to SSQ after the evaluation session (the first four questions of 16 questions in SSQ). (**b**) Graphical user interface for scoring the level of cybersickness corresponding to each VR scene during the evaluation session.

As depicted in Figure 3, the participants were asked to answer SSQ four times: one pre- and three post-evaluations. In contrast to the pre-assessment of SSQ acquired by handwriting, as shown in Figure 4a, each SSQ after the evaluation session was sent via a graphical user interface using a controller in the VR environment; thus, the participants did not need to take off their HMD.

#### 3.1.4. Scoring Cybersickness

Apart from SSQ scores, we also collected subjective cybersickness scores for each VR scene. Here, the goal is to analyze the finer-level content factor and to use it as a label in the cybersickness prediction scenario. To rate the degree of cybersickness in each VR scene, we used a modified version of the absolute category rating methodology [62]. As depicted in Figure 3, at each evaluation session, the participants were asked to assign the sickness score based on a five-point Likert-like scale: 5 = extreme sickness, 4 = strong sickness, 3 = sickness, 2 = mild sickness, and 1 = comfortable. The subjective scores were collected using a controller without taking off an HMD, as depicted in Figure 4b.

### 3.2. Acquisition of Physiological Signals

**Figure 5:**
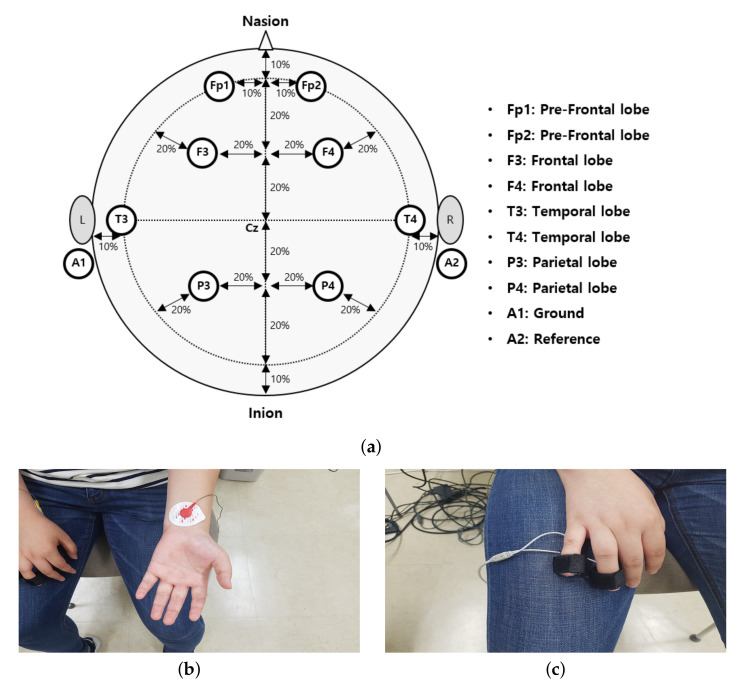
Electrode locations for obtaining biological measurements. (**a**) Eight EEG electrodes on scalp are located according to the international 10–20 system [65]. (**b**) Single ECG electrodes are placed on the wrists. (**c**) Two electrodes for GSR are placed on the fingers.

To objectively measure cybersickness, we acquired three types of physiological data during the experiment: EEG, ECG, and GSR. The LAXTHA’s PolyG-I system was used, which supports eight channels of EEG, single-channel ECG, and single-channel GSR with a 256 Hz sampling rate [63,64]. As depicted in Figure 5a, the EEG electrodes were located according to the international 10–20 system [65]. We obtained eight channels of brain signals: two on the pre-frontal lobes (Fp1 and Fp2), two on the frontal lobes (F3 and F4), two on the temporal lobes (T3 and T4), and two on the parietal lobes (P3 and P4). The impedance of each electrode was less than 5 KΩ. When measuring EEG signals, we applied the notch filters with 60 and 120 Hz stop-bands to reject electrical interferences before analog-to-digital conversion. As shown in Figure 5b,c, the electrodes for ECG and GSR are located at both wrists and the ring and little fingers. Physiological signals were recorded over an entire CYRE content. Those were divided into signals of suitable duration, and they were synchronized with each corresponding VR scene.

### 3.3. Participants and Environment

In the subjective evaluation, 203 volunteers participated (normal and corrected-to-normal vision; age range: 14 to 59 years; mean age: 27.58 years). The participants were divided into four groups: young men (under 30 years), middle-aged/elderly men (over 30 years), young women (under 30 years), and middle-aged/elderly women (over 30 years). The number of participants in each group is presented in Table 2. This categorization was designed to evaluate the effects of sex and age on cybersickness severity [66,67,68]. There were no special criteria for participation; however, in accordance with institutional review board recommendations, only people of a certain age range (except for children) without the underlying disease could participate. The participants were randomly recruited by a poster and an advertisement in online communities where a small fee was paid for a participant. All participants were non-experts in the fields related to VR and vision research. No cues relating to the study hypothesis were given during the instruction session before the evaluation. The time for subjective evaluation varied from person to person, but on average it took about three hours per person including instruction, filling out a consent form, and arranging system settings as depicted in Figure 3. The total period to complete the experiment was four months. In the laboratory environment, we used HTC VIVE as an HMD. A high-end workstation with Intel i7 8700 CPU, nVidia GTX1080Ti GPU, and 64 GB RAM was used to maintain an approximately 90 fps rendering rate; the goal was to minimize the motion-to-photon latency and the other hardware-oriented factors related to cybersickness. The data from a single participant were collected in a single sitting. The participants sat on chairs and were instructed to keep their heads stationary to ensure that reliable physiological signals were obtained.

During the study, 15 participants (11 women (mean age: 38.45) and four men (mean age: 37.25)) dropped out of the evaluation procedure owing to physical symptoms (vomit, nausea, and dizziness) and their own will, and the data of 18 participants who met two of the conditions below because of insincere ratings were rejected:

- the same scores for all 116 ratings,
- the discrepancy between the answers in consent and personal information, and
- the eye tracker capture showed the eyes were closed throughout the experiment.

The data of 16 participants were removed because of an evaluation system error. Finally, the data of 154 valid participants were determined as a valid dataset for statistical tests and cybersickness prediction. The details of the number of participants in each group after refinement are presented in Table 2.

Additional analysis of the participants who dropped out based on their individual and demographic characteristics may be possible; however, our study focuses on the VR content factors. Thus, the following analysis only deals with data from the 154 valid participants.

## 4. Experimental Results

### 4.1. Scene Factors and Cybersickness

We investigated the statistical relationship between each content factor and the cybersickness score to understand which types of content factors are crucial for provoking cybersickness. All participants were asked to rate all scenes as stated in Section 3.1.4, thus we calculated the mean opinion score (MOS) for the scenes with the same attributes and perform a paired *t*-test on them. The threshold significance level was set to 5%, and the outliers were removed based on the IQR rule at each factor of analysis. The flagged levels of significance *, **, and *** represent that the *p*-value is less than 0.05, 0.01, and 0.001, respectively. Here, significance values were adjusted with the Bonferroni correction for multiple tests.

#### 4.1.1. Camera Rotation

**Figure 6:**
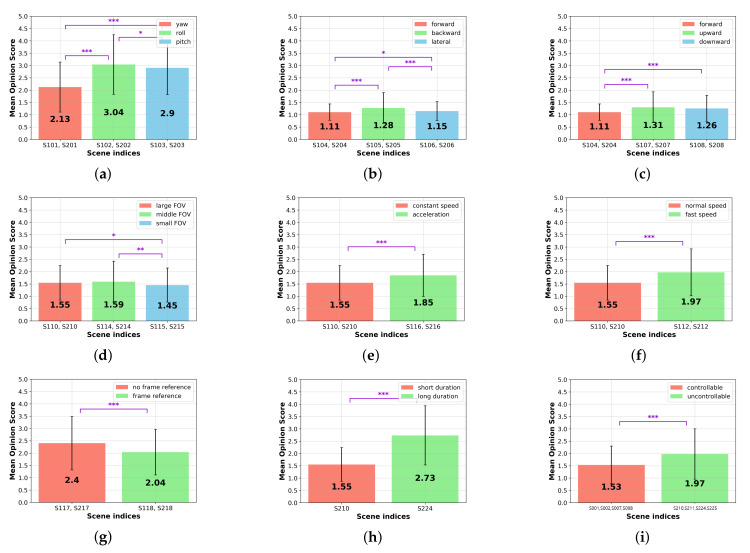
MOS comparisons of each VR content factor: (**a**) camera rotation, (**b**) horizontal camera translation, (**c**) vertical camera translation, (**d**) size of FOV, (**e**) translation acceleration, (**f**) translation speed, (**g**) frame reference, (**h**) duration, and (**i**) controllability. *, **, and *** represent that *p*-value is less than 0.05, 0.01, and 0.001, respectively.

The degree of cybersickness was analyzed depending on different uniaxial camera rotations: yaw $r_{y}$ (S101 and S201), roll $r_{r}$ (S102 and S202), and pitch $r_{p}$ (S103 and S203) scenes. As shown in Figure 6a, we found a statistical significance: MOS of $r_{r}$ scenes (mean: 3.039) is 43.0% and 4.7% higher than those of the $r_{y}$ (mean: 2.127, $p=3.35\times 10^{-34}<0.001$ ***) and $r_{p}$ scenes (mean: 2.903, p=0.040<0.05 *). Moreover, the results show that the MOS of $r_{p}$ scenes was 36.5% higher than for $r_{y}$ scenes ($p=1.25\times 10^{-39}<0.001$ ***). Overall, $r_{r}$ induces extreme cybersickness rather than the other camera rotations. Thus, drastic roll rotation has to be avoided in VR content.

#### 4.1.2. Camera Translation

We investigated which type of camera translation causes higher cybersickness. The obtained MOSs of the forward $t_{f}$ (S104 and S204), backward $t_{b}$ (S105 and S205), and lateral $t_{l}$ (S106 and S206) movement scenes were compared, as shown in (b). The results demonstrate that the cybersickness severity of $t_{b}$ (mean: 1.276) is significantly higher than those of the movements of $t_{f}$ (mean: 1.107, $p=4.44\times 10^{-7}<0.001$ ***) and $t_{l}$ (mean: 1.149, $p=7.49\times 10^{-5}<0.001$ ***).

Moreover, the $t_{f}$ scenes were compared to vertical translations: the upward $t_{u}$ (S107 and S207) and downward $t_{d}$ (S108 and S208) movement scenes. The result is depicted in Figure 6c. The MOSs of the $t_{u}$ and $t_{d}$ scenes are significantly higher than the $t_{f}$ scenes: 17.9% (mean: 1.305, $p=2.69\times 10^{-10}<0.001$ ***) and 15.3% (mean: 1.259, $p=5.95\times 10^{-10}<0.001$ ***), respectively.

The MOS of the $t_{u}$ scene is slightly higher than the downward scene; however, the difference is insignificant (p=0.108). Although there are statistically significant relationships between each different translational movement, overall MOS values are relatively low. Thus, a simple translational camera movement causes less cybersickness than the other VR content factors.

#### 4.1.3. FOV

We compared different sizes of FOV to investigate how much the restricted FOV is effective in reducing cybersickness. As shown in Figure 6d, there is no statistical significance between the large (S110 and S210) and the middle (S114 and S214) sizes of FOV scenes. Thus, we concluded that the marginal reduction of FOV is trivial to decrease the level of cybersickness. However, when the FOV is sufficiently smaller (S115 and S215), it significantly reduces the degree of cybersickness ∼6% (mean: 1.455) in comparison with the large (mean: 1.545, p=0.0305<0.05 *) and middle FOV (mean: 1.594, p=0.0012<0.01 **) scenes. Such results are consistent with well-known previous studies on FOV [21,46].

#### 4.1.4. Translation Acceleration

As shown in Figure 6e, the participants rated that the translation acceleration scenes (S116 and S216) made them experience 19.7% higher cybersickness rather than viewing the constant-speed scenes (S110 and S210) ($p=1.948\times 10^{-11}<0.001$ ***). This result is consistent with previous research [39].

#### 4.1.5. Translation Speed

In this paper, we compared cybersickness in moderate-speed (S110 and S210) and fast-speed translation scenes (S112 and S212), as shown in Figure 6f. The MOSs of the fast-speed translation scenes are significantly higher (27.5%) than those of the moderate-speed translation scenes ($p=1.417\times 10^{-16}<0.001$ ***). This result shows that not only translation acceleration, but also translation speed, is a factor that increases cybersickness.

#### 4.1.6. Frame Reference

To investigate how much the frame reference can alleviate cybersickness, the obtained MOSs of S117, S217, S118, and S218 were compared. The results show that the cybersickness severity is reduced by 15.2% when the frame reference is included rather than excluded ($p=1.927\times 10^{-11}<0.001$ ***), as shown in Figure 6g. We concluded that the deployment of the frame reference is recommended for producing VR content, forcing the unanticipated camera movement to the viewers to decrease cybersickness.

#### 4.1.7. Duration

In Figure 6h, we observed a statistically significant difference between cybersickness severities when the experience duration and the path length were extended. The results show that the longer scene (S224) induces increased cybersickness ($p=9.957\times 10^{-31}<0.001$ ***), compared with the short scene (S210).

#### 4.1.8. Controllability

To quantify how much cybersickness can be suppressed by the user’s intention with the navigation control, we compared the MOSs of the controllable (S001, S002, S007, and S008) and uncontrollable (S210, S211, S224, and S225) scene pairs whose attributes are identical except for controllability, thus the rest of the variables are controlled. As shown in (i), when controllability is given, the MOS is reduced by 22.5% in comparison with viewing an uncontrollable scene ($p=6.063\times 10^{-45}<0.001$ ***).

### 4.2. Physiological Signals and Cybersickness

This subsection aims to identify the cybersickness-related physiological patterns in the measured data. Each obtained EEG, ECG, and GSR signal was pre-processed, and then the extracted features were statistically analyzed in terms of cybersickness severity.

#### 4.2.1. Feature Processing

To filter the noise and remove artifacts contained in EEG, we applied a band-pass filter with a range of 1–59 Hz. Noninvasive electrodes were used to measure electrical activity on the human scalp. In general, the blind source separation is a critical problem to be resolved due to various artifacts including eye blinks and muscle movements. To address this, we applied independent component analysis to identify the linear projections maximizing the mutual independencies of the estimated components, which is a useful approach to isolate artifacts and brain activity [34]. From the signals decomposed by applying the independent component analysis, a single component with the maximum kurtosis was subtracted, which was regarded as a potential artifact [69]. After the pre-processing, we estimated the power spectral density for six waves with different frequency bands: delta (1–3.99 Hz), theta (4–7 Hz), alpha (8–15 Hz), beta (16–31 Hz), gamma (32–59 Hz), and Mu (8–12 Hz) waves. Here, the relative ratio of power spectral density was calculated for statistical analysis.

For ECG, we applied the de-trending filter to the raw data to eliminate the baseline-offset fluctuation. The QRS complex was detected by the peak detection algorithm. Three time-domain parameters were extracted for cybersickness analysis: beats per minute (BPM), the standard deviation of normal-to-normal intervals (SDNN), and the root mean square of the successive R-R interval difference (RMSSD).

For GSR, we applied the moving average filter to remove interferences. The mean amplitude over the VR scene duration was estimated as a cybersickness representation.

#### 4.2.2. Statistical Analysis

To understand the representative features associated with cybersickness, we conducted a three-step statistical analysis. For this analysis, the extracted features were grouped, along with the cybersickness scores obtained by the subjective evaluation; thus, five groups were composed. First, under the assumption that the features represent significant patterns according to the sickness level, we performed a one-way analysis of variance (ANOVA) on the five groups. Second, when ANOVA showed significant results within the selected feature, we performed an independent *t*-test on every combination of group pairs subsequently. Finally, we utilized the ordinary least squares (OLS) regression to observe whether the features varied monotonically following the level of cybersickness or not. The threshold significance level was set to 5%. Table 3 shows *p*-values from the statistical analysis. Here, significance values were adjusted with the Bonferroni correction for post hoc tests. Hence, the statistically insignificant features through ANOVA and *t*-tests were rejected.

#### 4.2.3. Discussion

**Figure 7:**
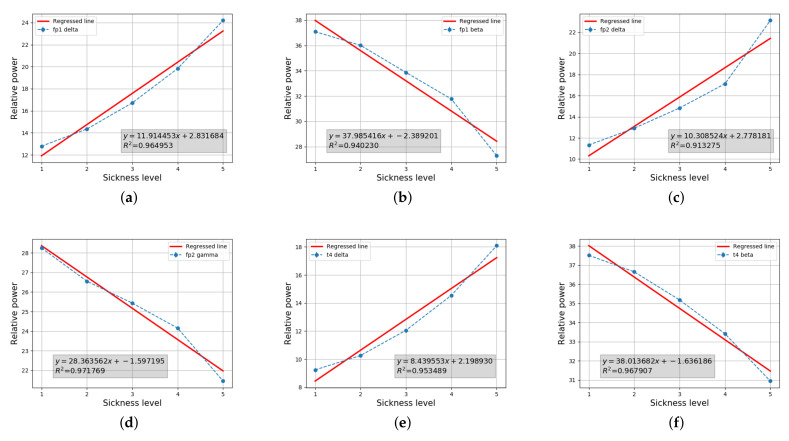
Selected features of EEG (i.e., relative power spectral density) through statistical tests and the results of a linear regression onto cybersickness scores. (**a**) Fp1 delta, (**b**) Fp1 beta, (**c**) Fp2 delta, (**d**) Fp2 gamma, (**e**) T4 delta, and (**f**) T4 beta.

Our results show that the six EEG features (i.e., relative power spectral densities of Fp1 delta, Fp1 beta, Fp2 delta, Fp2 gamma, T4 delta, and T4 beta) were determined as the valid patterns representing the level of cybersickness. Figure 7 shows that the cybersickness severity (horizontal axis) and EEG features (vertical axis) are highly correlated, given the coefficient of determination $R^{2}$ being greated than 0.9. The intercept and regressed coefficient values obtained by OLS regression are represented in the legend. The results in Figure 7a–d demonstrate that a variation of the relative power spectral density of the pre-frontal lobes is significantly related to the degree of cybersickness. This is reasonable, because it is well known that the prefrontal area is activated for the prior expectation of motion direction [70]. A large portion of designed CYRE content aims to artificially induce a conflict between anticipation and percipation, as explained in Bos’s model. Hence, this result suggests that a visual motion imposes a heavy load on the related neurological motion expectation process. Notably, the relative delta power of the overall area increases significantly when cybersickness increases, as shown in Table 3. As discovered in a previous study, a delta power increasing in various brain regions reflects a stress component affected by motion sickness [71]. Therefore, a similar analysis might be applied: cybersickness induced by viewing a VR scene can be treated as a stressification. Figure 7e shows that the relative delta power of the T4 lobes is also increased when the cybersickness level increases; this result is consistent with a previous physiological study [1]. In general, activities in the temporal lobe are involved in the sensory process to derive the appropriate retention of visual memory [72]. Thus, we can determine that a visually induced cybersickness is associated with the neural store mechanism of visual stimuli. The relative beta powers of Fp1 and T4 decrease significantly when the cybersickness increases, as shown in Figure 7b,f. According to Valentino and Dufresne’s demonstration [73], beta power variation reflects the process of attending to the stimuli of the task. Thus, according to our results, increases of cybersickness are accompanied by a decrease in the participants’ concentration. Although we could not find any statistical significance between GSR, ECG, and cybersickness severity in this study, recent work reported that such biological information could be the feature to predict the degree of cybersickness [74]. Such kinds of regression approaches are emerging based on machine learning techniques, and then it is expected that those factors would also be considered to predict potential cybersickness severity.

### 4.3. Individual Characteristics and Cybersickness

Beyond the VR content factors, the individual characteristics and sickness susceptibility might be related to the degree of cybersickness [59,60]. To investigate this, we analyzed the collected MSSQ and SSQ data by performing statistical tests.

#### 4.3.1. Sex and Cybersickness

First, we investigated whether sex determined cybersickness severity or not. For this, the participants were categorized into two groups: women and men, as shown in Table 2, and the independent two-sample *t*-test was performed on the pre-assessment of the SSQ and the three SSQ results after the participants had viewed the controllable urban ($C_{\mathrm{CU}})$, uncontrollable astrospace ($C_{\mathrm{UA}}$), and uncontrollable urban ($C_{\mathrm{UU}}$) scenes. As shown in Table 4, there is no significant difference in the cybersickness symptoms experienced by the women and men. This finding is consistent with a previous study of 36 participants [66]. In contrast, Koslucher et al. showed that the ratio of motion sickness incidence for women versus men was greater than 4:1 [67]. Such results suggest that sex is one of the future topics to be examined further as a part of the human factor for determining the severity of cybersickness.

#### 4.3.2. Age and Cybersickness

**Figure 8:**
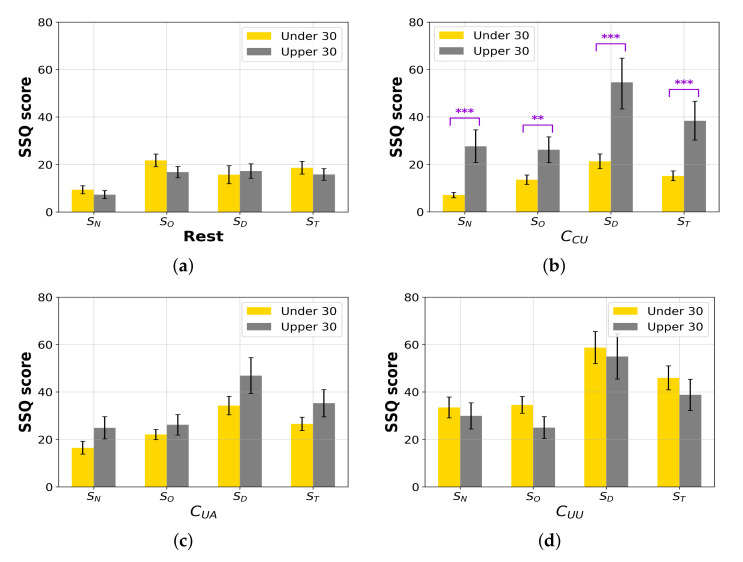
SSQ score comparison in accordance with age. (**a**–**d**) show the SSQ scores $s_{N}$, $s_{O}$, $s_{D}$, and $s_{T}$ of the rest state, $C_{\mathrm{CU}}$, $C_{\mathrm{UA}}$ and $C_{\mathrm{UU}}$, respectively. ** and *** represent that *p*-value is less than 0.01 and 0.001, respectively.

We hypothesized that age is a factor reflecting the individual characteristics of the experienced level of cybersickness. The participants were categorized into two groups as tabulated in Table 2: young and middle-aged [68]. We compared the age groups to discover a relationship between age and cybersickness. The independent two-sample *t*-test was performed on SSQ scores. Table 5 and Figure 8a–d show the results of the rest state and three-scene categories ($C_{\mathrm{CU}}$, $C_{\mathrm{UA}}$, and $C_{\mathrm{UU}}$). According to our results, there are statistical significances for every SSQ score of the $C_{\mathrm{CU}}$ scenes between the young and middle-aged groups. The results indicate that the elder participants experienced 393%, 193%, 256%, and 254% more severe nausea, disorientation, oculomotor fatigue, and total sickness from controllable content, respectively. Therefore, cybersickness is independent of age when viewing the general VR content. However, the proficiency in controlling is highly associated with sickness, because we observed that the middle-aged (upper 30) participants experienced more difficulties in controlling the VR scenes during the subjective evaluation, although the pre-training time had been given in the tutorial session as depicted in Figure 3.

#### 4.3.3. Susceptibility and Cybersickness

Based on the collected MSSQ and SSQ scores, we investigated how individual characteristics in motion sickness susceptibility affect the degree of cybersickness. For this analysis, the participants were categorized into the upper, middle, and lower 1/3 percentile groups of $s_{MSSQ,A}$, $s_{MSSQ,B}$, and $s_{MSSQ}$ susceptibility scores, respectively.

**Figure 9:**
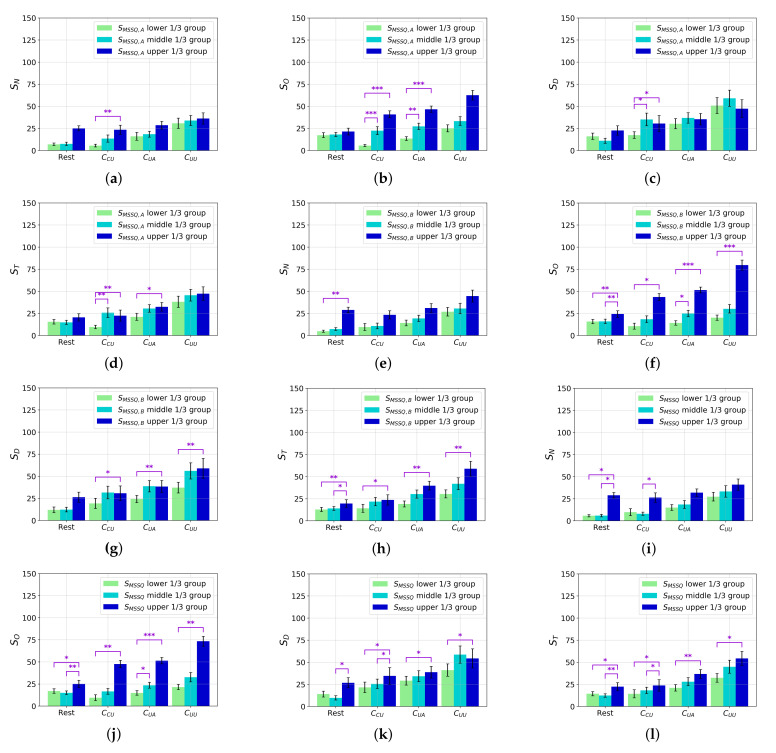
SSQ score comparison: individual sickness susceptibility and the scene categories. The three treatment groups (lower, middle, and upper 1/3 percentiles of susceptibility) are composed based on $s_{\mathrm{MSSQ},A}$ (**a**–**d**), $s_{\mathrm{MSSQ},B}$ (**e**–**h**), and $s_{\mathrm{MSSQ}}$ (**i**–**l**), respectively. *, **, and *** represent that *p*-value is less than 0.05, 0.01, and 0.001, respectively.

Figure 9 shows the results of the independent two-sample *t*-test performed on the SSQ scores. Figure 9a–d show the $s_{N}$, $s_{O}$, $s_{D}$, and $s_{T}$ scores of the three groups based on the $s_{MSSQ,A}$. We observe that sickness experience in childhood is related to the $C_{\mathrm{CU}}$ scenes across all SSQ scores. The $s_{MSSQ,A}$ upper 1/3 group experiences significantly more severe cybersickness than the $s_{MSSQ,A}$ lower 1/3 group for these scenes. For the $C_{\mathrm{UA}}$ scenes, we observe significantly higher $s_{O}$ and $s_{T}$ scores of the $s_{MSSQ,A}$ upper 1/3 group than those of the $s_{MSSQ,A}$ lower 1/3 group.

Figure 9e–h show the SSQ results based on the $s_{MSSQ,B}$ score. The results show that the sickness experience of adults was more sensitively reflected in cybersickness. The $s_{O}$, $s_{D}$, and $s_{T}$ values of the $s_{MSSQ,B}$ upper 1/3 group are significantly higher than those of the $s_{MSSQ,B}$ lower 1/3 group in all of the scene categories. However, in the case of $s_{N}$, no relationship is observed between previous sickness experience and nausea caused by viewing the VR scenes.

Figure 9i–l show the relationship between motion sickness susceptibility, $s_{MSSQ}$, and SSQ scores. All of the SSQ scores of the $s_{MSSQ}$ upper 1/3 group are significantly higher than those of the $s_{MSSQ}$ lower 1/3 group, except for $s_{N}$, after viewing uncontrollable scenes.

Despite some exceptions in the statistical results, an individual’s susceptibility for motion sickness is mostly correlated with cybersickness. Moreover, the general cybersickness symptoms when viewing a controllable scene strongly depend on motion sickness susceptibility. Consequently, motion sickness susceptibility is a major individual factor related to cybersickness. These results might be considered when providing the VR services, for example, embedding a certain interface that pre-determines the difficulty level of VR games based on the obtained $s_{MSSQ}$ from an arbitrary user.

### 4.4. Cybersickness Prediction

After obtaining the cybersickness score for each VR scene during the evaluation session, this subjective score can be used as the label corresponding to the VR scene for the supervision of the cybersickness prediction model. Thus, apart from the previous schemes that employed SSQ results as labels of long-term content [26,75], more data-label pairs (i.e., VR scene and cybersickness score) are available to train the predictor, which might yield a more reliable performance. To show how this constructed dataset can quantify the level of cybersickness, we extracted simple visual features from the VR scenes. These features were regressed onto the subjective score by using support vector regression.

**Figure 10:**
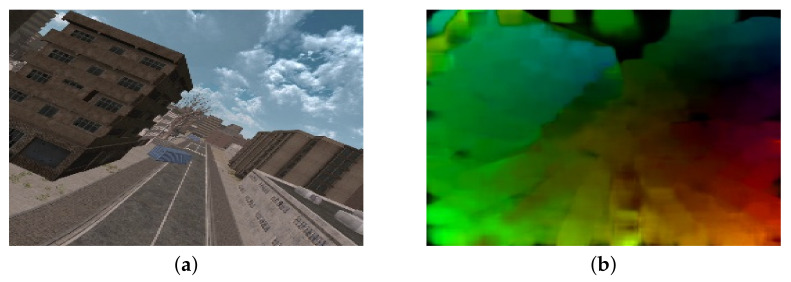
Motion vector visualization. (**a**,**b**) are the rendered VR scene and the estimated motion vector, respectively.

As stated in Section 4.1, a scene movement is an important contributing factor of cybersickness; these results are also consistent with Bos’s theory. Sensory conflict remains a hypothetical parameter that cannot be measured directly, but which is driven by visual stimuli (i.e., visual motion) in a virtual environment [76]. In this context, to extract representative features that reflect visual motion, we used an optical flow algorithm [77]. Then, the motion vector was estimated as shown in Figure 10. Let ${\vec{m}}_{n,t}$ be the motion vector of the nth pixel at the tth frame. The first visual feature $f_{1}$ is an average magnitude of motion, which is obtained by the spatio-temporal mean pooling of ${\vec{m}}_{n,t}$ in the VR scene:

(2) $$f_{1}=\frac{1}{T\cdot N}\sum_{t}\sum_{n}\left|{\vec{m}}_{n,t}\right|,$$

where *N* and *T* are the total number of motion vectors and frames, respectively. Moreover, we assumed that the holistic distribution of motion vectors is an effective visual feature that represents the perception of self-motion. Thus, the variance of motion magnitude was extracted:

(3) $$f_{2}=\frac{1}{T\cdot N}\sum_{t}\sum_{n}{\left(\left|{\vec{m}}_{n,t}\right|-f_{1}\right)}^{2}.$$

Additionally, we utilized the pth percentile-pooling method, which is a well-known technique reflecting the human visual system or the human perceptual characteristic that the most severe spatial visual stimulus has a dominant effect on the overall delivered quality experience, which was employed in several studies related to quantifying the quality of experience fields [78,79,80,81,82]. In this paper, we captured such characteristics as the visual features by calculating the average and variance for both the upper and lower pth percentiles of motion vectors (we set p=10%):

(4) $$\begin{array}{cc} \; & f_{3}=\frac{1}{T\cdot N/10}\sum_{t}\sum_{n\in n_{p,u}}\left|{\vec{m}}_{n,t}\right|, \end{array}$$

(5) $$\begin{array}{cc} \; & f_{4}=\frac{1}{T\cdot N/10}\sum_{t}\sum_{n\in n_{p,u}}{\left(\left|{\vec{m}}_{n,t}\right|-f_{3}\right)}^{2}, \end{array}$$

(6) $$\begin{array}{cc} \; & f_{5}=\frac{1}{T\cdot N/10}\sum_{t}\sum_{n\in n_{p,l}}\left|{\vec{m}}_{n,t}\right|, \end{array}$$

(7) $$\begin{array}{cc} \; & f_{6}=\frac{1}{T\cdot N/10}\sum_{t}\sum_{n\in n_{p,l}}{\left(\left|{\vec{m}}_{n,t}\right|-f_{5}\right)}^{2}, \end{array}$$

where ${\left|{\vec{m}}_{t}\right|}_{p,u}$ and ${\left|{\vec{m}}_{t}\right|}_{p,l}$ denote the upper and lower pth $\left|{\vec{m}}_{n,t}\right|$s; thus,$n_{p,u}=\left\{n|\left|{\vec{m}}_{n,t}\right|>{\left|{\vec{m}}_{t}\right|}_{p,u}\right\}$ and $n_{p,l}=\left\{n|\left|{\vec{m}}_{n,t}\right|<{\left|{\vec{m}}_{t}\right|}_{p,l}\right\}$, respectively.

Using (2)–(7), the above-defined six visual features were extracted from the motion vectors and compared with the learned model using support vector regression along with the subjective cybersickness score. For this analysis, the visual features were learned by the cross-validation method with 100 train-test trials. For each trial, the randomly chosen training set consisted of 80% of the constructed dataset, i.e., 6396 (123participants×52scenes) data-label pairs, and the test set consists of the remaining 20%, i.e., 1612 (31participants×52scenes) data-label pairs. The predictive performance was evaluated using the Spearman’s rank-order correlation coefficient (SROCC) and the Pearson’s linear correlation coefficient (PLCC) relative to the cybersickness score.

Table 6 shows the performance of the tested models in terms of SROCC and PLCC. To better understand the contributions of each extracted visual feature, we compared the performance of the subsets of $\left(f_{1},f_{2}\right)$, $\left(f_{1},f_{2},f_{3},f_{4}\right)$, $\left(f_{1},f_{2},f_{5},f_{6}\right)$, and $\left(f_{1},f_{2},f_{3},f_{4},f_{5},f_{6}\right)$. Although simple visual features were extracted, the prediction model shows reasonable performance. Note that when $f_{1}$–$f_{4}$ (i.e., the mean and variance of all of the vectors and the upper pth percentiles of motion vector) were employed to train the model, we achieved the best predictive performance. Otherwise, when the mean and variance of the lower pth percentiles of the motion vectors were added as the visual features $f_{5}$ and $f_{6}$, the predictive performance decreased. The result shows that a stronger visual motion stimulates a higher degree of sensory conflicts, which provokes severe cybersickness.

Moreover, to verify that the constructed dataset is also valid for the previous methods of cybersickness prediction, we compared three previously developed cybersickness prediction schemes: Padmanaban et al., Kim et al. [61], and Kim et al. [27]. The first and second schemes are regression methods based on hand-crafted features, and the third model captures cybersickness-related representations automatically via supervised learning with a deep neural network. Table 7 shows the performance of previous schemes in terms of SROCC and PLCC. Notably, the deep learning approach could achieve reliable predictive performance with our dataset without any data augmentation since we constructed a massive dataset with 8008 (154 participants × 52 scenes) labels. Our results imply that the constructed dataset is applicable in various scenarios, including amelioration, suppression, and prediction of cybersickness.

### 4.5. General Discussion

Our results show that the lower-level attributes of the CYRE content that were considered are significantly related to the degree of cybersickness. In particular, camera movement, including rotation, translation, navigating speed, and acceleration, is a dominant factor that determines cybersickness incidence and its severity (Figure 6). Due to the fact that the environmental setting is designed to evaluate visually induced cybersickness, such results conclude that CYRE content and the dataset are well constructed to reflect previous neurological theories in terms of sensory conflict. Additionally, the hand-crafted features of brain signals (Fp1 delta, Fp1 beta, Fp2 delta, Fp2 gamma, T4 delta, and T4 beta) effectively represent the level of cybersickness (Table 3 and Figure 7); this implies that an advanced cybersickness monitoring system can be achieved by employing these features as biomarkers in various VR therapy scenarios. Moreover, we observed that some human factors are also related to cybersickness in terms of individual characteristics: age (Table 5 and Figure 8) and sickness susceptibility (Figure 9). This work also shows the possibility of quantification/objectification of the cybersickness level based on substantial data-label pairs (Table 6 and Table 7); this would lead to the development of advanced data-driven approaches for modeling an accurate cybersickness predictor.

## 5. Conclusions

In this study, we created CYRE content with 52 scenes reflecting different cybersickness factors. For these VR scenes, we collected subjective opinions and objective data using a protocol designed for cybersickness evaluation. Using the constructed dataset, we analyzed cybersickness in terms of each factor. We verified that a large number of data-label pairs could be used in supervised learning to produce a reliable cybersickness prediction. Although we showed a simple cybersickness predictor based on the rendered VR scene, it would also be possible to train the predictor based on biological signals since our dataset contains biological data-label pairs. The presented results are limited by a part of the analysis, compared with the considerable amount of data that would ensure that a number of combinations of variables are available to be analyzed; furthermore, an additional in-depth study might be available. We expect that the constructed dataset and the reported results can be applied to create improved VR services since this work is in parallel with the IEEE standard association activities [83]. Additionally, the results can be applied to the development of the next-generation visual content targeted to the extended reality and holography beyond VR. In particular, this study shows a direction to quantitatively analyze the more subjective and psychological quality of experience (including presence, attentiveness, emotion, and aesthetic quality) in terms of visual content and services in conjunction with statistics and learning-based methods. To achieve this, we are now attempting to generalize and quantify immersion in accordance with these additional human factors.

## Figures and Tables

**Table 1:** List of scenes and their corresponding attributes in CYRE content.

Scene Index	Background	Camera				FoV	Frame Reference	Duration	Controllability	Scene Category
		Movement	Rotation and Translation ^1^	Translation Acceleration	Translation Speed					
S001	Urban	Complex	$\left(t_{f},r_{r},r_{y}\right)$	No	Moderate (4 m/s)	Large ($90^{\circ }$)	No	Short (∼14.6 s)	Controllable	$C_{\mathrm{CU}}$ (∼144.8 s)
S002	Urban	Complex	$\left(t_{f},t_{u},t_{d},r_{r},r_{y},r_{p}\right)$	No	Moderate (4 m/s)	Large ($90^{\circ }$)	No	Short (∼14.9 s)	Controllable	
S003	Urban	Complex	$\left(t_{f},t_{u},t_{d},r_{r},r_{y},r_{p}\right)$	No	Moderate (4 m/s)	Large ($90^{\circ }$)	No	Short (∼14.8 s)	Controllable	
S004	Urban	Complex	$\left(t_{f},t_{u},t_{d},r_{r},r_{y},r_{p}\right)$	No	Moderate (4 m/s)	Large ($90^{\circ }$)	No	Short (∼14.2 s)	Controllable	
S005	Urban	Complex	$\left(t_{f},t_{u},t_{d},r_{r},r_{y},r_{p}\right)$	No	Moderate (4 m/s)	Large ($90^{\circ }$)	No	Short (∼15.6 s)	Controllable	
S006	Urban	Complex	$\left(t_{f},t_{u},t_{d},r_{r},r_{y},r_{p}\right)$	No	Moderate (4 m/s)	Large ($90^{\circ }$)	No	Short (∼15.2 s)	Controllable	
S007	Urban	Complex	$\left(t_{f},r_{r},r_{y}\right)$	No	Moderate (4 m/s)	Large ($90^{\circ }$)	No	Long (∼28.0 s)	Controllable	
S008	Urban	Complex	$\left(t_{f},r_{r},r_{y}\right)$	No	Moderate (4 m/s)	Large ($90^{\circ }$)	No	Long (∼27.5 s)	Controllable	
S101	Astrospace	Simple	$r_{y}$	No	No translation	Large ($90^{\circ }$)	No	Short (13 s)	Uncontrollable	
S102	Astrospace	Simple	$r_{r}$	No	No translation	Large ($90^{\circ }$)	No	Short (13 s)	Uncontrollable	$C_{\mathrm{UA}}$ (234 s)
S103	Astrospace	Simple	$r_{p}$	No	No translation	Large ($90^{\circ }$)	No	Short (13 s)	Uncontrollable	
S104	Astrospace	Simple	$t_{f}$	No	Moderate (4 m/s)	Large ($90^{\circ }$)	No	Short (13 s)	Uncontrollable	
S105	Astrospace	Simple	$t_{b}$	No	Moderate (4 m/s)	Large ($90^{\circ }$)	No	Short (13 s)	Uncontrollable	
S106	Astrospace	Simple	$t_{l}$	No	Moderate (4 m/s)	Large ($90^{\circ }$)	No	Short (13 s)	Uncontrollable	
S107	Astrospace	Simple	$t_{u}$	No	Moderate (4 m/s)	Large ($90^{\circ }$)	No	Short (13 s)	Uncontrollable	
S108	Astrospace	Simple	$t_{d}$	No	Moderate (4 m/s)	Large ($90^{\circ }$)	No	Short (13 s)	Uncontrollable	
S109	Astrospace	Complex	$\left(t_{f},t_{u},t_{d},r_{p}\right)$	No	Moderate (4 m/s)	Large ($90^{\circ }$)	No	Short (13 s)	Uncontrollable	
S110	Astrospace	Complex	$\left(t_{f},r_{r},r_{y}\right)$	No	Moderate (4 m/s)	Large ($90^{\circ }$)	No	Short (13 s)	Uncontrollable	
S111	Astrospace	Complex	$\left(t_{f},t_{u},t_{d},r_{r},r_{y},r_{p}\right)$	No	Moderate (4 m/s)	Large ($90^{\circ }$)	No	Short (13 s)	Uncontrollable	
S112	Astrospace	Complex	$\left(t_{f},r_{r},r_{y}\right)$	No	Fast (9.2 m/s)	Large ($90^{\circ }$)	No	Short (13 s)	Uncontrollable	
S113	Astrospace	Complex	$\left(t_{f},t_{u},t_{d},r_{r},r_{y},r_{p}\right)$	No	Fast (9.2 m/s)	Large ($90^{\circ }$)	No	Short (13 s)	Uncontrollable	
S114	Astrospace	Complex	$\left(t_{f},r_{r},r_{y}\right)$	No	Moderate (4 m/s)	Middle ($45^{\circ }$)	No	Short (13 s)	Uncontrollable	
S115	Astrospace	Complex	$\left(t_{f},r_{r},r_{y}\right)$	No	Moderate (4 m/s)	Small ($30^{\circ }$)	No	Short (13 s)	Uncontrollable	
S116	Astrospace	Complex	$\left(t_{f},r_{r},r_{y}\right)$	Yes (160%) ^2^	Moderate (4 m/s)	Large ($90^{\circ }$)	No	Short (13 s)	Uncontrollable	
S117	Astrospace	Complex	$\left(t_{f},t_{u},t_{d},r_{r},r_{y},r_{p}\right)$	Yes (160%)	Moderate (4 m/s)	Large ($90^{\circ }$)	No	Short (13 s)	Uncontrollable	
S118	Astrospace	Complex	$\left(t_{f},t_{u},t_{d},r_{r},r_{y},r_{p}\right)$	Yes (160%)	Moderate (4 m/s)	Large ($90^{\circ }$)	Yes	Short (13 s)	Uncontrollable	
S201	Urban	Simple	$r_{y}$	No	No translation	Large ($90^{\circ }$)	No	Short (13 s)	Uncontrollable	
S202	Urban	Simple	$r_{r}$	No	No translation	Large ($90^{\circ }$)	No	Short (13 s)	Uncontrollable	$C_{\mathrm{UU}}$ (371 s)
S203	Urban	Simple	$r_{p}$	No	No translation	Large ($90^{\circ }$)	No	Short (13 s)	Uncontrollable	
S204	Urban	Simple	$t_{f}$	No	Moderate (4 m/s)	Large ($90^{\circ }$)	No	Short (13 s)	Uncontrollable	
S205	Urban	Simple	$t_{b}$	No	Moderate (4 m/s)	Large ($90^{\circ }$)	No	Short (13 s)	Uncontrollable	
S206	Urban	Simple	$t_{l}$	No	Moderate (4 m/s)	Large ($90^{\circ }$)	No	Short (13 s)	Uncontrollable	
S207	Urban	Simple	$t_{u}$	No	Moderate (4 m/s)	Large ($90^{\circ }$)	No	Short (13 s)	Uncontrollable	
S208	Urban	Simple	$t_{d}$	No	Moderate (4 m/s)	Large ($90^{\circ }$)	No	Short (13 s)	Uncontrollable	
S209	Urban	Complex	$\left(t_{f},t_{u},t_{d},r_{p}\right)$	No	Moderate (4 m/s)	Large ($90^{\circ }$)	No	Short (13 s)	Uncontrollable	
S210	Urban	Complex	$\left(t_{f},r_{r},r_{y}\right)$	No	Moderate (4 m/s)	Large ($90^{\circ }$)	No	Short (13 s)	Uncontrollable	
S211	Urban	Complex	$\left(t_{f},t_{u},t_{d},r_{r},r_{y},r_{p}\right)$	No	Moderate (4 m/s)	Large ($90^{\circ }$)	No	Short (13 s)	Uncontrollable	
S212	Urban	Complex	$\left(t_{f},r_{r},r_{y}\right)$	No	Fast (9.2 m/s)	Large ($90^{\circ }$)	No	Short (13 s)	Uncontrollable	
S213	Urban	Complex	$\left(t_{f},t_{u},t_{d},r_{r},r_{y},r_{p}\right)$	No	Fast (9.2 m/s)	Large ($90^{\circ }$)	No	Short (13 s)	Uncontrollable	
S214	Urban	Complex	$\left(t_{f},r_{r},r_{y}\right)$	No	Moderate (4 m/s)	Middle ($45^{\circ }$)	No	Short (13 s)	Uncontrollable	
S215	Urban	Complex	$\left(t_{f},r_{r},r_{y}\right)$	No	Moderate (4 m/s)	Small ($30^{\circ }$)	No	Short (13 s)	Uncontrollable	
S216	Urban	Complex	$\left(t_{f},r_{r},r_{y}\right)$	Yes (160%)	Moderate (4 m/s)	Large ($90^{\circ }$)	No	Short (13 s)	Uncontrollable	
S217	Urban	Complex	$\left(t_{f},t_{u},t_{d},r_{r},r_{y},r_{p}\right)$	Yes (160%)	Moderate (4 m/s)	Large ($90^{\circ }$)	No	Short (13 s)	Uncontrollable	
S218	Urban	Complex	$\left(t_{f},t_{u},t_{d},r_{r},r_{y},r_{p}\right)$	Yes (160%)	Moderate (4 m/s)	Large ($90^{\circ }$)	Yes	Short (13 s)	Uncontrollable	
S219	Urban	Complex	$\left(t_{f},r_{r},r_{y}\right)$	No	Moderate (4 m/s)	Large ($90^{\circ }$)	No	Short (13 s)	Uncontrollable	
S220	Urban	Complex	$\left(t_{f},r_{r},r_{y}\right)$	No	Moderate (4 m/s)	Large ($90^{\circ }$)	No	Short (13 s)	Uncontrollable	
S221	Urban	Complex	$\left(t_{f},r_{r},r_{y}\right)$	No	Moderate (4 m/s)	Large ($90^{\circ }$)	No	Short (13 s)	Uncontrollable	
S222	Urban	Complex	$\left(t_{f},r_{r},r_{y}\right)$	No	Moderate (4 m/s)	Large ($90^{\circ }$)	No	Short (13 s)	Uncontrollable	
S223	Urban	Complex	$\left(t_{f},r_{r},r_{y}\right)$	No	Moderate (4 m/s)	Large ($90^{\circ }$)	No	Short (13 s)	Uncontrollable	
S224	Urban	Complex	$\left(t_{f},r_{r},r_{y}\right)$	No	Moderate (4 m/s)	Large ($90^{\circ }$)	No	Long (24 s)	Uncontrollable	
S225	Urban	Complex	$\left(t_{f},r_{r},r_{y}\right)$	No	Moderate (4 m/s)	Large ($90^{\circ }$)	No	Long (24 s)	Uncontrollable	
S226	Urban	Complex	$\left(t_{f},r_{r},r_{y}\right)$	Yes (160%)	Moderate (4 m/s)	Large ($90^{\circ }$)	No	Long (24 s)	Uncontrollable	

^1^*r_y_*, *r_r_*, and *r_p_* represent yaw, roll, and pitch rotations, and *t_f_*, *t_b_*, *t_l_*, *t_u_*, and *t_d_* represent forward, backward, lateral, upward, and downward translations, respectively. ^2^ The translation speed gradually increases up to 160% from the initial translation speed.

**Table 2:** The number of participants (total 154).

	Men	Women
Young	43	63
(under 30 yr)	(mean age 22.98 yr)	(mean age 21.02 yr)
Middle-aged	28	20
(upper 30 yr)	(mean age 38.82 yr)	(mean age 42.40 yr)

**Table 3:** Statistics concerning physiological features and cybersickness.

Data	Feature	*p*-Values of Statistical Test					Data	Feature	*p*-Values of Statistical Test				
		ANOVA	*t*-test (1, 2)	*t*-Test (2, 3)	*t*-Test (3, 4)	*t*-Test (4, 5)			ANOVA	*t*-Test (1, 2)	*t*-Test (2, 3)	*t*-Test (3, 4)	*t*-Test (4, 5)
EEG Fp1	Delta	<0.001 ***	<0.001 ***	<0.001 ***	<0.001 ***	<0.05 *	EEG T3	Delta	<0.001 ***	<0.001 ***	<0.001 ***	0.2240	<0.01 **
	Theta	<0.001 ***	<0.01 **	<0.05 *	0.8881	0.1415		Theta	<0.001 ***	<0.01 **	<0.01 **	0.5028	0.3645
	Alpha	<0.001 ***	<0.01 **	0.0700	0.5406	0.2676		Alpha	<0.001 ***	<0.001 ***	0.0848	0.9419	0.3362
	Beta	<0.001 ***	<0.01 **	<0.001 ***	<0.05 *	<0.01 **		Beta	<0.001 ***	<0.001 ***	<0.001 ***	0.1927	<0.05 *
	Gamma	<0.001 ***	<0.001 ***	<0.01 **	<0.01 **	0.2268		Gamma	<0.001 ***	<0.001 ***	<0.001 ***	0.5954	0.2294
	Mu	<0.001 ***	<0.01 **	0.0793	0.4439	<0.05 *		Mu	<0.001 ***	<0.001 ***	0.0790	0.8074	0.5842
EEG Fp2	Delta	<0.001 ***	<0.001 ***	<0.01 **	<0.05 *	<0.001 ***	EEG T4	Delta	<0.001 ***	<0.01 **	<0.001 ***	<0.01 **	<0.05 *
	Theta	<0.001 ***	<0.001 ***	<0.05 *	0.4147	<0.01 **		Theta	<0.001 ***	0.0531	0.0991	0.0718	0.1780
	Alpha	<0.001 ***	<0.001 ***	<0.05 *	0.4755	0.1357		Alpha	<0.001 ***	<0.01 **	0.1741	0.9474	0.6311
	Beta	<0.001 ***	<0.001 ***	<0.01 **	0.0625	<0.001 ***		Beta	<0.001 ***	<0.01 **	<0.001 ***	<0.05 *	<0.05 *
	Gamma	<0.001 ***	<0.001 ***	<0.01 **	<0.05 *	<0.01 **		Gamma	<0.001 ***	<0.001 ***	<0.01 **	0.1085	0.0813
	Mu	<0.001 ***	<0.001 ***	0.1113	0.2698	<0.05 *		Mu	<0.001 ***	<0.05 *	0.1957	0.6509	0.3801
EEG F3	Delta	<0.001 ***	<0.01 **	<0.001 ***	<0.05 *	0.1582	EEG P3	Delta	<0.001 ***	<0.01 **	<0.001 ***	0.3325	<0.01 **
	Theta	<0.01 **	0.9996	0.0539	0.1751	0.9101		Theta	<0.05 *	0.7200	<0.05 *	0.2063	0.4868
	Alpha	<0.01 **	0.0582	0.7701	0.3810	<0.01 **		Alpha	0.2211	0.2263	0.5675	0.6228	0.8228
	Beta	<0.001 ***	0.4779	<0.01 **	<0.05 *	<0.05 *		Beta	<0.001 ***	0.0578	<0.01 **	0.1048	<0.01 **
	Gamma	<0.001 ***	<0.01 **	<0.05 *	0.1821	0.1738		Gamma	<0.001 ***	<0.05 *	<0.01 **	0.3226	0.7697
	Mu	<0.05 *	0.3330	0.4663	0.5991	<0.01 **		Mu	0.1596	0.3706	0.5515	0.2886	0.9481
EEG F4	Delta	<0.001 ***	<0.001 ***	<0.001 ***	0.1333	<0.01 **	EEG P4	Delta	<0.001 ***	0.1651	<0.001 ***	0.1990	<0.01 **
	Theta	<0.001 ***	0.0537	0.0663	0.4235	0.3859		Theta	0.0510	0.1515	0.0258	0.8868	0.3862
	Alpha	<0.001 ***	<0.01 **	0.3938	0.5183	0.1883		Alpha	<0.05 *	0.1066	0.6538	0.5482	0.1261
	Beta	<0.001 ***	<0.05 *	<0.001 ***	0.1960	<0.01 **		Beta	<0.001 ***	0.3294	<0.01 **	0.3998	<0.001 ***
	Gamma	<0.001 ***	<0.001 ***	<0.05 *	0.2301	0.0701		Gamma	<0.001 ***	0.1040	<0.05 *	0.2021	0.1609
	Mu	<0.001 ***	<0.05 *	0.1889	0.6718	<0.05 *		Mu	<0.01 **	0.4021	0.8191	0.3378	0.0573
ECG	BPM	<0.01 **	0.1308	0.2590	0.9342	0.0910	GSR	Mean	<0.01 **	0.1110	0.1162	0.6136	0.2411
	SDNN	<0.001 ***	<0.001 ***	0.7875	0.8688	<0.01 **							
	RMSSD	<0.001 ***	<0.001 ***	0.7840	0.9575	<0.01 **							

*, **, and *** represent that *p*-value is less than 0.05, 0.01, and 0.001, respectively.

**Table 4:** Statistics concerning sex and cybersickness.

		$s_{N}$	$s_{O}$	$s_{D}$	$s_{T}$
Rest	Men	7.325	17.190	13.423	14.960
	Women	9.817	14.630	18.358	19.947
	*p*-value	0.336	0.138	0.311	0.191
$C_{\mathrm{CU}}$	Men	14.140	19.627	35.794	24.844
	Women	12.582	15.490	27.840	20.055
	*p*-value	0.745	0.353	0.352	0.432
$C_{\mathrm{UA}}$	Men	20.273	26.395	44.991	33.059
	Women	17.974	20.763	32.480	26.017
	*p*-value	0.631	0.156	0.081	0.183
$C_{\mathrm{UU}}$	Men	35.945	34.110	63.386	47.952
	Women	29.450	29.551	52.856	40.327
	*p*-value	0.356	0.429	0.345	0.351

**Table 5:** Statistics concerning age and cybersickness.

		$s_{N}$	$s_{O}$	$s_{D}$	$s_{T}$
Rest	Under 30	9.321	21.694	15.680	18.571
	Upper 30	7.281	16.756	17.217	15.747
	*p*-value	0.466	0.213	0.771	0.494
$C_{\mathrm{CU}}$	Under 30	7.018	13.505	21.280	15.132
	Upper 30	27.616	26.131	54.581	38.384
	*p*-value	<0.001 ***	<0.01 **	<0.001 ***	<0.001 ***
$C_{\mathrm{UA}}$	Under 30	16.448	22.043	34.240	26.524
	Upper 30	24.854	26.131	46.888	35.235
	*p*-value	0.102	0.342	0.103	0.127
$C_{\mathrm{UU}}$	Under 30	33.445	34.502	58.720	45.912
	Upper 30	35.235	29.875	54.947	38.778
	*p*-value	0.639	0.124	0.755	0.419

** and *** represent that *p*-value is less than 0.01 and 0.001, respectively.

**Table 6:** Performance of 100 trials of randomly chosen training and test sets.

Visual Features	SROCC	PLCC
$\left(f_{1},f_{2}\right)$	0.635	0.602
$\left(f_{1},f_{2},f_{3},f_{4}\right)$	0.665	0.642
$\left(f_{1},f_{2},f_{5},f_{6}\right)$	0.603	0.574
$\left(f_{1},f_{2},f_{3},f_{4},f_{5},f_{6}\right)$	0.606	0.602

**Table 7:** Performance of 100 trials by features of the previous schemes.

	SROCC	PLCC
Padmanaban et al. [26]	0.532	0.577
Kim et al. [61]	0.621	0.581
Kim et al. [27]	0.733	0.780

## Data Availability

Not applicable.
